# Nicotinamide Mononucleotide Is Safely Metabolized and Significantly Reduces Blood Triglyceride Levels in Healthy Individuals

**DOI:** 10.7759/cureus.28812

**Published:** 2022-09-05

**Authors:** Shintarou Kimura, Misa Ichikawa, Suzuka Sugawara, Tomoko Katagiri, Yuumi Hirasawa, Takahiro Ishikawa, Wataru Matsunaga, Akinobu Gotoh

**Affiliations:** 1 Environmental Science, StateArt Incorporated, Tokyo, JPN; 2 Gene Therapy, StateArt Incorporated, Tokyo, JPN; 3 Anti-Aging, StateArt Incorporated, Tokyo, JPN; 4 Agriculture, StateArt Incorporated, Tokyo, JPN; 5 Biochemistry, StateArt Incorporated, Tokyo, JPN; 6 Basic Medicine, StateArt Incorporated, Tokyo, JPN; 7 Gene Therapy, Joint-Use Research Facilities, Hyogo Medical University, Nishinomiya, JPN; 8 Gene Therapy, Department of Education for Medical Research Base, Hyogo Medical University, Nishinomiya, JPN

**Keywords:** anti-aging, obesity, sirtuin 1, triglyceride, nicotinamide adenine dinucleotide, nicotinamide mononucleotide

## Abstract

An increase in nicotinamide adenine dinucleotide (NAD^+^) levels alleviates age-related disease progression and promotes healthy life expectancy. Several studies have demonstrated that NAD^+ ^levels can be efficiently replenished via nicotinamide mononucleotide (NMN) intake; additionally, the safety of its oral ingestion has been confirmed in recent clinical trials. However, the efficacy and safety of intravenous NMN administration in humans remain unclear. Therefore, we verified its safety in 10 healthy volunteers. Intravenous administration of NMN did not affect electrocardiograms, pulse, and blood pressure, nor did it affect metabolic markers in the liver, heart, pancreas, and kidneys. These results indicate that intravenous NMN administration is safe and beneficial in humans. Furthermore, NMN administration significantly increased blood NAD^+^ levels without damaging blood cells and significantly reduced blood triglyceride (TG) levels. These findings imply that intravenous administration of NMN may lead to the prevention and treatment of diseases associated with increased TG levels, such as fatty liver and diabetes.

## Introduction

The risk of type II diabetes mellitus, Alzheimer-type dementia, and cardiomegaly increases with age [1-3]; therefore, the suppression of aging is sought after to prevent aging-related disorders. For this purpose, the use of nicotinamide (NAM) adenine dinucleotide (NAD^+^) is attracting attention [4]. NAD^+^ works as a substrate for sirtuins, poly (ADP-ribose) polymerases (PARP), and cluster of differentiation 38 (CD38) [5]. Although NAD^+ ^is digested by these enzymes to produce NAM, NAM is acted upon by nicotinamide phosphoribosyltransferase (NAMPT) and converted to nicotinamide mononucleotide (NMN) so that NAD^+^ is resynthesized via the salvage pathway [6]. NAMPT is the rate-limiting enzyme in NAD^+^ synthesis, and its expression decreases with age [7]. Therefore, in recent years, NMNs have attracted attention as NAD^+^ precursors.

In a recent study, Mills et al. orally administered 300 mg/kg NMN to 5-17 month-old mice for one year, which resulted in a 9% weight loss despite higher dietary intake than that in the control group; they found that not only was the aging-related increase in cholesterol level suppressed but there were also no significant changes in aging-related gene expression in the skeletal muscle, liver, and fat [8]. Recently, this group conducted clinical trials on oral administration of NMN in healthy individuals, demonstrating that NMN is safely metabolized in the blood without affecting glucose metabolism, lipid metabolism, liver, kidneys, and leukocytes [9]. Based on their research reports, several clinics in Japan have begun to offer intravenous administration of NMN as a preventive measure against aging; although the safety of transient oral administration of NMN has been proven in humans [9], that of intravenous administration has not been confirmed. Intravenous administration poses a higher risk of damage to major organs, such as the heart, pancreas, and kidneys than oral administration, because the nutrients and drugs administered circulate directly in the blood without going through the first-pass effect of the liver [10], the centre of detoxification. However, based on the findings of these studies, we speculated that NMN could be safely administered intravenously, as it is a metabolite produced in the body [11]. In addition, in previous studies, no adverse events were observed with the intravenous administration of NAD^+^ to humans [12]. Mammalian cells import NAD^+^ precursors for intracellular NAD^+^ synthesis, as NAD^+^ cannot cross the plasma membrane via passive diffusion because of its hydrophilicity, positive charge, and molecular size. Consequently, using NAD^+^, which cannot penetrate the cell membrane, is unsuitable [13] as no dedicated transporter has been identified in mammals. Therefore, NAD^+^ is degraded into extracellular NAD^+^-consuming enzymes, such as CD38, CD73, and CD153, and then intracellularly incorporated into the cell with a transporter specific for NAD^+^ precursors [12]. It has been suggested that NAM produced by the degradation of NAD+ through CD38 is synthesized into NMN by extracellular NAMPT and incorporated intracellularly [4]. Therefore, we decided to investigate whether NMN is safely metabolized in humans after transient intravenous administration and is capable of increasing intracellular NAD^+^ levels.

NAD^+ ^has been studied as a coenzyme required for activation of the sirtuin protein family members, which have an anti-aging function [14]. Among the members of the sirtuin family, sirtuin 1 (SIRT1) has been studied as a factor that directly prevents aging via glucose metabolism [15], insulin secretion [16], lipid metabolism [17], angiogenesis [18], and elimination of reactive oxygen species (ROS) [19]. Therefore, we evaluated NAD+ and SIRT1 activities in blood cells after the intravenous administration of NMN.

## Materials and methods

Study population

**Figure 1:**
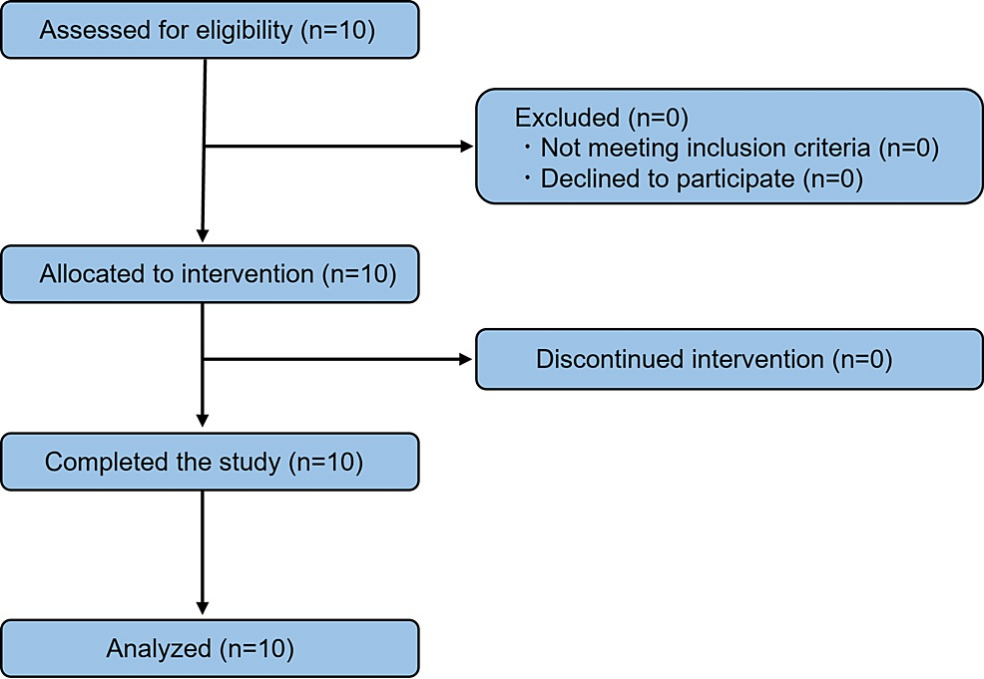
Flow diagram of the clinical trial.

The administration was done starting from May 31, 2021, sample collection was conducted from June to July 2021, and data analysis was completed on September 30, 2021. NMN was purchased from Nordeste (Tokyo, Japan). We conducted an open-label, single-arm exploratory study on 10 healthy individuals (Figure 1), including five males and five females (age, 20-70 years), recruited from the Tokyo Tsukishima Clinic. This study was approved by the Japanese Organization for Safety Assessment of Clinical Research (#20210623-02; 23/06/2021) and registered with the University Hospital Medical Information Network (UMIN; Japan) (UMIN-ID: UMIN000047134; 09/03/2022). This article was previously posted to the Research Square preprint server on January 31, 2022 (https://doi.org/10.21203/rs.3.rs-1298321/v1). The subjects provided written informed consent before starting the clinical trial. The present study was conducted in accordance with the Declaration of Helsinki and Ethical Guidelines for Medical and Health Research Involving Human Subjects set by the Japanese Ministry of Health, Labor, and Welfare. Individuals with a history of disease diagnosis, malignant neoplasms, serious infections, psychiatric disorders, ophthalmic disorders, allergic disorders, and metabolic disorders were excluded from the study.

The subjects fasted for 12 h before intravenous administration of NMN until the end of the clinical trial but were provided free hydration. Intravenous drip infusion was performed at 5 mL/min by dissolving 300 mg of NMN in 100 mL of saline and inserting an extension tube through a vein in the middle of the arm. The weights of the subjects were measured, and chest radiographs were obtained before and after the intravenous administration of NMN. General disorders and administration site conditions were diagnosed by a physician, according to Common Terminology Criteria for Adverse Events (CTCAE) v5.0-Japan Clinical Oncology Group (JCOG), before and after the intravenous administration of NMN. Body temperature, blood pressure, pulse, and oxygen saturation were measured at 0.5, 1, 2, 3, and 5 h before and after the intravenous administration of NMN, and blood was collected concomitantly. Blood collection tubes containing EDTA-2Na (ethylenediaminetetraacetic acid disodium; Falco Biosystems, Kyoto, Japan) were used to obtain 17 mL of blood samples, and 12 mL of the collected blood was sent to Falco Biosystems for hematological analysis. The remaining blood was stored at −80 °C and used to measure blood NAD^+^ levels and SIRT1 activation. Urine was collected 1, 3, and 5 h before and after the intravenous administration of NMN to the subjects; urinary urobilinogen, protein, glucose, pH, and occult blood levels were measured at the Tokyo Tsukishima Clinic.

NAD^+^/NADH assay

The amounts of NAD^+^ and NADH in blood cells were assessed using the NAD/NADH Assay Kit-WST (Dojindo Laboratories, Kumamoto, Japan). Briefly, a 20 µL blood sample was dissolved in 300 µL extraction buffer, and 250 µL of the sample extract was transferred to an MWCO 10 K filtration tube for ultrafiltration. The obtained filtrate was transferred to two new Eppendorf tubes (100 µL each), one of which was incubated at 60 °C for 1 h. The unheated sample was used as the total NAD^+^ sample, and the heated sample was used as the NADH sample. The absorbance of these samples was measured at 450 nm using a Varioskan plate reader (Thermo Fisher Scientific, Yokohama, Japan). The amount of NAD^+^ was calculated by subtracting the amount of NADH from the amount of total NAD^+^.

SIRT1 assay

Nuclear proteins were extracted from blood cells using the LysoPureTM Nuclear and Cytoplasmic Extractor Kit (FUJIFILM Wako Pure Chemical Corporation, Osaka, Japan). Briefly, 500 µL of blood was added to 500 µL of initial buffer, incubated on ice for 10 min, vortexed, and centrifuged at 500 × g for 10 min at 4 °C. The supernatant was transferred to a new Eppendorf tube as a cytosolic fraction; buffer 1 was added to the pellet again, and the pellet was vortexed and centrifuged at 500 × g for 10 min at 4 °C. The supernatant was discarded, and 250 µL of buffer 2 was added, after which the mixture was vortexed and then incubated on ice for 30 min. The supernatant was collected as a nuclear extraction fraction after centrifugation at 2000 × g for 10 min at 4 °C.

The cytoplasmic and nuclear fractions were evaluated for SIRT1 activation using CycLex® SIRT1/Sir2 Deacetylase Fluorometric Assay Kit Ver.2 (Medical & Biological Laboratories, Aichi, Japan). Briefly, 25 µL distilled water, 5 µL SIRT1 assay buffer, 5 µL fluoro-substrate peptide, and 5 µL developer were added to a 50 µL nuclear extraction and cytosol fraction samples, in this order, mixed well, and the reaction was initiated by adding 50 µL enzyme sample following incubation at 37 °C for 30 min before quenching the reaction using 20 µL stop solution. The prepared samples were analyzed using a Fluoroskan microplate fluorometer (Thermo Fisher Scientific, Japan) at an excitation wavelength of 350 nm and an emission wavelength of 450 nm to detect SIRT1 activity.

Quantitative reverse transcription polymerase chain reaction (RT-qPCR) analysis of *NAMPT*, *NANOG*, and *p16*

RNA was extracted from 250 µL of whole blood using RNAiso (Takara, Otsu, Japan). The Thunderbird SYBR qPCR/RT Set (Toyobo, Osaka, Japan) was used for reverse transcription and RT-qPCR. RT-qPCR was performed using the StepOnePlus™ real-time PCR system (Applied Biosystems, Foster City, CA, USA). The sequences of the primers used are listed in Table 1 [20-22]. For positive control, qPCR Human Reference cDNA and Total RNA (Takara, Otsu, Japan) was used. The cycling conditions were as follows: initial denaturation at 95 °C for 20 s, followed by 40 cycles of denaturation at 95 °C for 5 s, and annealing and elongation at 0 °C for 20 s. Melting curves were measured at 95 °C for 15 s, 60 °C for 1 min, and 95 °C for 15 s. The results obtained were quantified using the standard method and corrected with actin beta (ACTB) expression.

**Table 1:** RT-qPCR primer sequences RT-qPCR: Quantitative reverse transcription polymerase chain reaction; NAMPT: Nicotinamide phosphoribosyltransferase

Gene	Forward	Reverse	Reference
NAMPT	5'-GCCAGCAGGGAATTTTGTTA-3'	5'-TGATGTGCTGCTTCCAGTTC-3'	[20]
NANOG	5'-CATGAGTGTGGATCCAGCTTG-3'	5'-CCTGAATAAGCAGATCCATGG-3'	[21]
p16	5'-CTGTCCTGCGTGTTGAAAGA-3'	5'-TTGGGTAATTTTTGGGATCTACA-3'	[22]

Statistical analysis

All results are expressed as mean ± standard deviation. Statistically significant differences between RT-qPCR were analyzed using Student's t-test, and other statistically significant differences were analyzed using a one-way analysis of variance (ANOVA) with Bonferroni’s post-hoc test using the add-in software Statcel4 (Version 4.0; OMS Publishing, Inc., Tokorozawa, Japan). The significance levels were set at *p < 0.05 and **p < 0.01.

## Results

Analysis performed by a physician indicated that there were no abnormalities or general disorders in administration site conditions, urinalysis, electrocardiograms, and chest radiographs before and after the intravenous administration of NMN (Tables 2-4). There were no significant changes in body weight or body mass index before and 5 h after the intravenous administration of NMN.

**Table 2:** Demographic and clinical characteristics of healthy individuals after the intravenous administration of NMN. The results are expressed as mean ± standard deviation (n = 10). NMN: Nicotinamide mononucleotide.

Variable	Pre-treatment		Post-treatment	
Age (years)	43.4 (±12.6)		43.4 (±12.6)	
Weight (kg)	66.1 (±9.6)		65.7 (±9.6)	
Body mass index (kg/m^2^)	24.4 (±3.5)		24.3 (±3.4)	
Electrocardiogram	Normal		Normal	
Chest X-ray	Normal		Normal	

**Table 3:** Characteristics of subjects after NMN treatment for urinalysis. Results are expressed as mean ± standard deviation (n = 10). NMN: Nicotinamide mononucleotide.

Test item	Baseline	1 h	3 h	5 h
Urobilinogen	Normal	Normal	Normal	Normal
Proteins	Negative	Negative	Negative	Negative
Glucose	Negative	Negative	Negative	Negative
pH	6.00 (±0.77)	6.60 (±0.66)	6.70 (±0.64)	6.50 (±0.67)
Occult blood	Negative	Negative	Negative	Negative

**Table 4:** Physician toxicity evaluation according to CTCAE v5.0-JCOG after NMN treatment. Results are expressed as mean ± standard deviation (n = 10). NMN: Nicotinamide mononucleotide. CTCAE: Common Terminology Criteria for Adverse Events; JCOG: Japan Clinical Oncology Group

Characteristics	Pretreatment	Post-treatment
Chills	0 (±0)	0 (±0)
Edema face	0 (±0)	0 (±0)
Fatigue	0 (±0)	0 (±0)
Gait disturbance	0 (±0)	0 (±0)
Infusion site extravasation	0 (±0)	0 (±0)
Injection site reaction	0 (±0)	0 (±0)
Malaise	0 (±0)	0 (±0)
Pain	0 (±0)	0 (±0)

**Figure 2:**
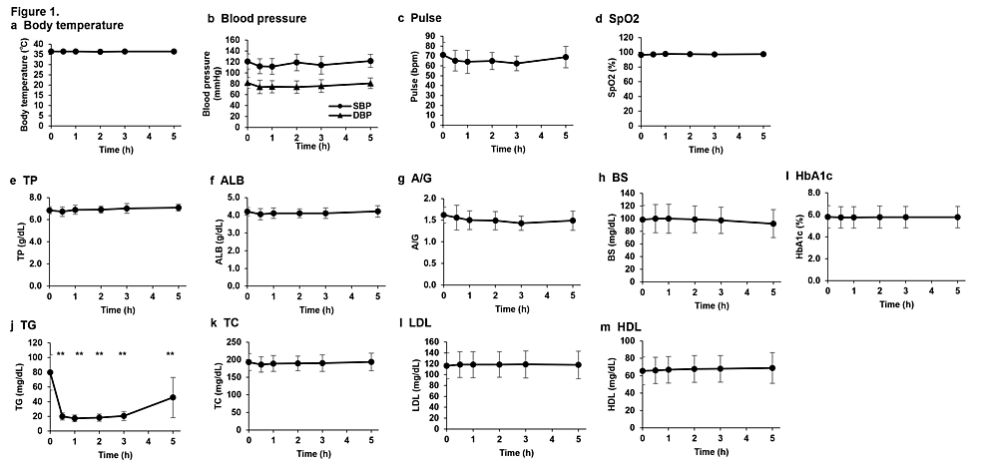
Changes in clinical parameters and sugar, lipid, and protein metabolism markers due to intravenous administration of NMN. (a) Body temperature, (b) blood pressure, (c) pulse, and (d) SpO2 were measured before and 0.5, 1, 2, 3, and 5 h after intravenous administration of NMN. Protein metabolism was evaluated in the plasma before and after the intravenous NMN administration based on (e) TP, (f) ALB, and (g) A/G; glucose metabolism was evaluated based on (h) BS and (i) HbA1c; lipid metabolism was evaluated based on (j) TG, (k) TC, (l) LDL, and (m) HDL. Data were analyzed via a one-way analysis of variance with Bonferroni’s post-test and expressed as mean ± standard deviation (n = 10, ** p < 0.01). NMN: Nicotinamide mononucleotide; TP: Total protein; ALB: Albumin; BS: Blood sugar; HbA1c: Hemoglobin A1C; TG: Triglyceride; TC: Total cholesterol; LDL: Low-density lipoprotein; HDL: High-density lipoprotein; SpO2: Oxygen saturation

Analyses of body temperature, systolic blood pressure, diastolic blood pressure, pulse, and oxygen saturation at 0.5, 1, 2, 3, and 5 h after the intravenous injection of NMN showed no significant differences in all parameters before and after administration (Figures 2a-2d). No significant effects on plasma protein levels and glucose metabolism were observed at 0.5, 1, 2, 3, and 5 h after the intravenous administration of NMN (Figures 2e-2i). Although LDL, HDL, and total cholesterol levels did not significantly differ before and 0.5, 1, 2, 3, and 5 h after the intravenous administration of NMN (Figures 2k-2m), triglyceride (TG) levels decreased significantly from 0.5 to 5 h after administration, and after 5 h, there was a slight tendency to return to the level before administration, notwithstanding a significant difference (Figure 2j).

**Figure 3:**
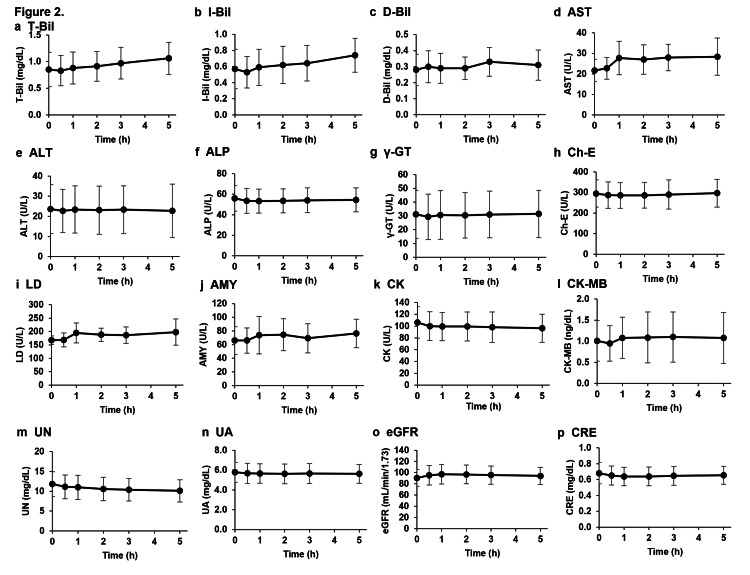
Effects of intravenous NMN administration on metabolic markers of liver, pancreas, heart, and kidney. Levels of metabolic markers of the liver, pancreas, heart, and kidney were measured in plasma obtained from subjects before and at 0.5, 1, 2, 3, and 5 h after intravenous NMN administration. The effects of the intravenous NMN administration on liver metabolism were evaluated via (a) T-Bill, (b) I-Bill, (c) D-Bill, (d) AST, (e) ALT, (f) ALP, (g) γ-GT, (h) Ch-E, and (i) LD; pancreatic metabolism was evaluated based on (j) AMY; cardiac metabolism was observed based on (k) CK and (l) CK-MB; renal metabolism was evaluated based on (m) UN, (n) UA, (o) eGFR, and (p) CRE. The results are expressed as mean ± standard deviation (n = 10). NMN: Nicotinamide mononucleotide; T-Bill: Total bilirubin; I-Bill: Indirect bilirubin; D-Bill: Direct bilirubin; AST: Aspartate transaminase; ALT: Alanine aminotransferase; ALP: Alanine phosphotransferase; γ-GT: Gamma-glutamyl transferase; LD: Lactate dehydrogenase; Ch-E: Cholinesterase; AMY: Amylase; CK: Creatine kinase; CK-MB: CK myocardial band; UN: Urea nitrogen; CRE: Creatinine; UA: Urinalysis; eGFR: Estimated glomerular filtration rate

**Figure 4:**
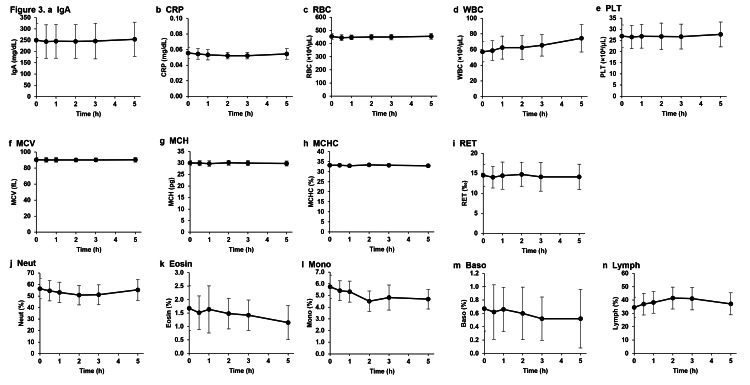
Effects of intravenous NMN administration on immune marker levels and blood cells. Immune markers (a) IgA and (b) CRP were measured in plasma obtained from patients before and 0.5, 1, 2, 3, and 5 h after the intravenous NMN administration. Blood cells, red blood cell markers, and leukocyte fractions were analyzed in blood obtained from patients before and 0.5, 1, 2, 3, and 5 h after the intravenous NMN administration. The damage to blood cells was measured based on the number of (c) RBC, (d) WBC, and (e) PLT; the effect of NMN administration on erythrocytes was observed based on (f) MCV, (g) MCH, (h) MCHC, and (i) RET; the effect of NMN administration on the leukocyte fraction was observed in (j) Neut, (k) Eosin, (l) Mono, (m) Basso, and (n) Lymph. The results are expressed as mean ± standard deviation (n = 10). NMN: Nicotinamide mononucleotide; IgA: Immunoglobulin A; CRP: C-reactive protein; RBC: Red blood cell; WBC: White blood cell; PLT: Platelet; MCV: Mean corpuscular volume; MCH: Mean corpuscular hemoglobin; MCHC: Mean corpuscular hemoglobin concentration; RET: Reticulocyte; Neut: Neutrophil; Eosin: Eosinophil; Mono: Monocyte; Basso: Basophil; Lymph: Lymphocyte

We investigated the pharmacokinetics specific to metabolic markers of the liver, heart, pancreas, and kidneys after the intravenous administration of NMN because drugs administered intravenously can rapidly reach organs throughout the body and may burden specific organs even if the substances are safe for oral administration. Liver-, pancreas-, heart-, and kidney-related metabolism marker levels in the plasma measured 0.5, 1, 2, 3, and 5 h after the intravenous injection of NMN showed no significant differences when compared to those before administration (Figure 3). Furthermore, analyses of red blood cells, white blood cells, platelets, and related markers in the blood showed no significant changes before and at 0.5, 1, 2, 3, and 5 h after the intravenous administration of NMN (Figure 4).

**Figure 5:**
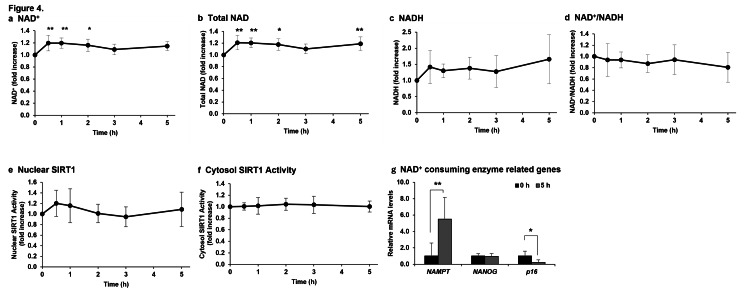
Effect of NMN administration on increased NAD+ level and SIRT1 activation. The amount of NAD+ and the activity of SIRT were measured in blood obtained from patients before and 0.5, 1, 2, 3, and 5 h after intravenous administration of NMN (a-f). NAD+-consuming enzyme-related genes were measured in blood cells before and 5 h after the intravenous administration of NMN (g). The results were analyzed via a one-way analysis of variance with Bonferroni’s post-test and are expressed as mean ± standard deviation (n = 10, * p < 0.05, ** p < 0.01). NMN: Nicotinamide mononucleotide; NAD+: Nicotinamide adenine dinucleotide; NADH: Reduced nicotinamide adenine dinucleotide; SIRT1: Sirtuin 1

Next, the total amount of NAD^+^ and NADH, and the ratio of NAD^+^ to NADH in the blood were measured to determine whether intravenously administered NMN could efficiently increase the amount of NAD^+^ in blood cells (Figure 5). NAD+/NADH blood levels measured 0.5, 1, 2, 3, and 5 h after the intravenous NMN administration showed a significant increase in NAD^+^ levels from 0.5 to 3 h compared to those before administration (Figure 5a) and a significant increase in total NAD^+^ level except 4 h after administration (Figure 5b). In contrast, NADH levels and the NAD^+^/NADH ratio could not be measured accurately because the measured NADH values varied considerably (Figures 5c, 5d).

To suppress aging, NAD^+^ should be utilized by sirtuin family proteins as a coenzyme during an increase in its levels [14]; therefore, we sought to determine whether SIRT1, which is considered to be particularly important for aging, is activated via intravenous administration of NMN. The activation of nuclear SIRT1 also increased, similar to the trend of NAD^+^ synthesis, after the intravenous NMN administration (Figure 5e); however, no significant difference was observed because of the large variation in the measured values. Cytosolic SIRT1 activation showed almost no change before and after the intravenous administration of NMN (Figure 5f).

Genes transcriptionally regulated by SIRT1, PARP1, and CD38 activation in blood cells were analyzed via RT-qPCR before and 5 h after the intravenous administration of NAD^+^ (Figure 5g). In the present study, of all the genes whose mRNA transcription was altered via SIRT1 activation, *NAMPT* was selected. The mRNA expression of *NAMPT *was significantly increased after the intravenous administration of NMN. *NANOG* was selected from the list of genes whose transcription is altered via PARP1 activation. The mRNA expression of *NANOG* did not change before or after the intravenous administration of NMN. *p16* was selected from the list of genes whose transcription is altered via activation of CD38. The mRNA expression of *p16 *was significantly reduced after the administration of NMN compared with that before the intravenous administration of NMN.

## Discussion

NMN, which is a precursor of NAD^+^, has been proven to increase NAD^+^ levels via uptake into the body in both rodents and humans [9,23]. Although NMN has been studied using various administration methods, such as oral, intraperitoneal injection, and intravenous injection, to enhance NAD^+^ synthesis [7], the efficacy and safety of intravenous administration in humans have not been verified. Our study showed that a single intravenous dose of 300 mg of NMN enhanced NAD^+^ activity in blood cells without affecting the levels of erythrocytes, leukocytes, and platelets, nor those of major markers in the liver, heart, pancreas, and kidneys.

**Figure 6:**
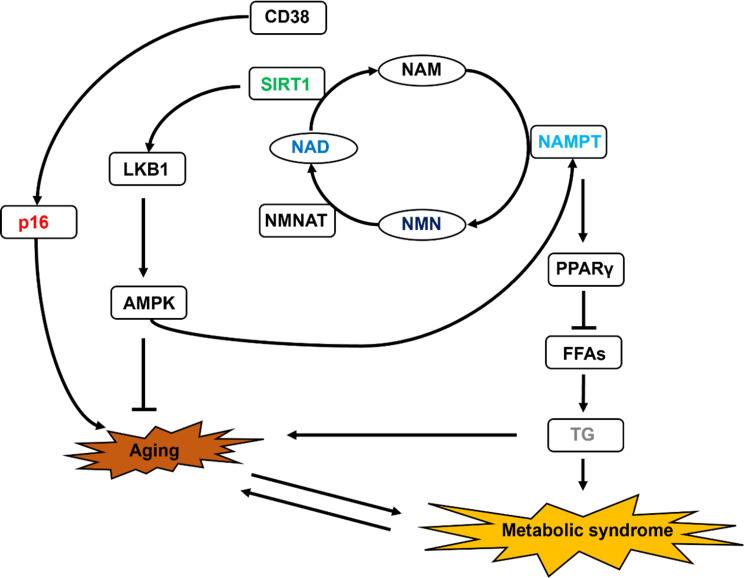
Schematic showing the mechanism of NMN metabolism. CD38: Cluster of differentiation 38; NAD: Nicotinamide adenine dinucleotide; NAM: Nicotinamide; NAMPT: Nicotinamide phosphoribosyltransferase; NMN: Nicotinamide mononucleotide; NMNAT: Nicotinamide mononucleotide adenylyltransferase; SIRT1: Sirtuin 1

Unexpectedly, TG levels were significantly reduced after the intravenous administration of NMN without affecting low-density lipoprotein (LDL) levels in the blood. No reduction in TG levels was observed in previous clinical trials upon oral administration of NMN [9], suggesting that NMN may be metabolized via different pathways when administered orally and intravenously. This is thought to be caused by NMN being metabolized in cells throughout the body by avoiding the first passage through the liver via intravenous administration. A previous study reported that the increase in TG levels in the plasma and liver of mice deficient in adipocyte-specific NAMPT1, an enzyme that synthesizes NMN from NAM, indicates that increased intracellular NMN synthesis plays a critical role in decreasing TG levels [24]. We hypothesize that the SIRT1 activated by NAD^+^ synthesized via NAMPT in adipocytes regulates adiponectin expression through deacetylation of PPARγ (peroxisome proliferator-activated receptor gamma) (Figure 6). Adiponectin in adipocytes reduces the amount of free fatty acids (FFAs) released into the blood; subsequently, FFAs in the blood are taken up by the liver, and TG is synthesized [25]. The activation of PPARγ by NAMPT leads to the suppression of TG synthesis in the blood. The markedly elevated mRNA expression of *NAMPT* in blood cells in the present study suggests that similar phenomena may occur in adipocytes. It is extremely difficult to conclude that there was no change in blood cholesterol levels in the present study. The blood clearance of LDL requires the enhanced expression of LDL receptors on the cell membrane surface [26]. LDL protein expression might not have increased 5 h after the intravenous administration of NMN. Since the decrease in TG levels due to the intravenous administration of NMN is a very interesting phenomenon, we would like to investigate the detailed mechanism by conducting cell experiments in the future, including its relationship with LDL and HDL.

Increased intracellular NAD^+^ levels lead to SIRT1 activation [14], but an increase in nuclear SIRT1 expression required the same time as that for NAD^+^ expression, with no significant difference observed in the present study. This might have been because SIRT1 was already moderately activated, as the study was performed under fasting conditions (12 h before administration of NMN). We analyzed the genes whose expression was altered owing to SIRT1, PARP1, and CD38 activation since NAD^+^ is involved in activating CD38 and PARPs in addition to sirtuins [5]. Expectedly, the mRNA expression of *NAMPT* was increased in the blood cells after the intravenous administration of NMN. Therefore, we hypothesize that AMPK (AMP-activated protein kinase) is activated by SIRT1 via LKB1 deacetylation and that the intravenous administration of NMN increased the mRNA expression of *NAMPT* via this pathway. The expression of pluripotent stem cell markers, such as *NANOG*, is regulated by PARP1 [21]. No large fluctuations in the expression of the *NANOG* gene were observed, suggesting that PARP1 was not significantly activated even after the intravenous administration of NMN. Aging marker genes, such as* p16*, are regulated by CD38 [27]. The mRNA expression of *p16* in blood cells was reduced by intravenous administration of NMN; thus, it is suggested that CD38 may not be activated. In addition, *p16* is known as a marker gene that upregulates not only aging but also DNA damage, suggesting that intravenous administration of NMN is safe. To accurately determine whether the marker gene levels investigated this time were altered by the activation of SIRT1, PARP, and CD38, it is necessary to investigate the activation of PARP, CD38, and SIRT1 in the future.

## Conclusions

Our clinical study demonstrated that 300 mg NMN administration is tolerated by humans because it does not cause significant damage to blood cells, the liver, pancreas, heart, and kidneys when injected intravenously and effectively increases the amount of NAD+ in blood cells. Furthermore, the increase in intracellular and plasma levels of NAD+ and NAD+ metabolites, such as methyl nicotinamide (MNA), N-methyl-2-pyridone-5-carboxamide (2Py), and N-methyl-4-pyridone-5-carboxamide (4Py), by intravenous administration of NMN should be examined by precise analysis using liquid chromatography-mass spectrometry (LC-MS). In addition, the feasibility of intravenous administration of 300 mg NMN suggests that NAMPT may be activated and TG levels may be suppressed, which may help improve obesity and prevent aging. Since NAMPT levels are increased by intravenous administration of NMN, it is expected that NAD may be continuously synthesized via the salvage pathway with a single administration. Further studies should examine the effects of LDL and HDL as well as blood TG on long-term intravenous administration of NMN and the relationship between changes in these values and body weight and body fat percentage.

## References

[1] Khosla S, Farr JN, Tchkonia T, Kirkland JL (2020). The role of cellular senescence in ageing and endocrine disease. Nat Rev Endocrinol.

[2] Wissler Gerdes EO, Zhu Y, Weigand BM, Tripathi U, Burns TC, Tchkonia T, Kirkland JL (2020). Cellular senescence in aging and age-related diseases: Implications for neurodegenerative diseases. Int Rev Neurobiol.

[3] Yan M, Sun S, Xu K (2021). Cardiac aging: from basic research to therapeutics. Oxid Med Cell Longev.

[4] Covarrubias AJ, Perrone R, Grozio A, Verdin E (2021). NAD+ metabolism and its roles in cellular processes during ageing. Nat Rev Mol Cell Biol.

[5] Verdin E (2015). NAD⁺ in aging, metabolism, and neurodegeneration. Science.

[6] Yoshino J, Baur JA, Imai SI (2018). NAD+ intermediates: the biology and therapeutic potential of NMN and NR. Cell Metab.

[7] Yoshino J, Mills KF, Yoon MJ, Imai S (2011). Nicotinamide mononucleotide, a key NAD(+) intermediate, treats the pathophysiology of diet- and age-induced diabetes in mice. Cell Metab.

[8] Mills KF, Yoshida S, Stein LR (2016). Long-term administration of nicotinamide mononucleotide mitigates age-associated physiological decline in mice. Cell Metab.

[9] Irie J, Inagaki E, Fujita M (2020). Effect of oral administration of nicotinamide mononucleotide on clinical parameters and nicotinamide metabolite levels in healthy Japanese men. Endocr J.

[10] Pond SM, Tozer TN (1984). First-pass elimination. Basic concepts and clinical consequences. Clin Pharmacokinet.

[11] Schweiger M, Hennig K, Lerner F (2001). Characterization of recombinant human nicotinamide mononucleotide adenylyl transferase (NMNAT), a nuclear enzyme essential for NAD synthesis. FEBS Lett.

[12] Grant R, Berg J, Mestayer R, Braidy N, Bennett J, Broom S, Watson J (2019). A pilot study investigating changes in the human plasma and urine NAD+ metabolome during a 6 hour intravenous infusion of NAD. Front Aging Neurosci.

[13] Yang T, Chan NY, Sauve AA (2007). Syntheses of nicotinamide riboside and derivatives: effective agents for increasing nicotinamide adenine dinucleotide concentrations in mammalian cells. J Med Chem.

[14] Bonkowski MS, Sinclair DA (2016). Slowing ageing by design: the rise of NAD+ and sirtuin-activating compounds. Nat Rev Mol Cell Biol.

[15] Guarente L (2006). Sirtuins as potential targets for metabolic syndrome. Nature.

[16] Zabolotny JM, Kim YB (2007). Silencing insulin resistance through SIRT1. Cell Metab.

[17] Schug TT, Li X (2011). Sirtuin 1 in lipid metabolism and obesity. Ann Med.

[18] Potente M, Ghaeni L, Baldessari D (2007). SIRT1 controls endothelial angiogenic functions during vascular growth. Genes Dev.

[19] Salminen A, Kaarniranta K, Kauppinen A (2013). Crosstalk between oxidative stress and SIRT1: impact on the aging process. Int J Mol Sci.

[20] Nacarelli T, Lau L, Fukumoto T (2019). NAD+ metabolism governs the proinflammatory senescence-associated secretome. Nat Cell Biol.

[21] Wiśnik E, Płoszaj T, Robaszkiewicz A (2017). Downregulation of PARP1 transcription by promoter-associated E2F4-RBL2-HDAC1-BRM complex contributes to repression of pluripotency stem cell factors in human monocytes. Sci Rep.

[22] Takahashi A, Loo TM, Okada R (2018). Downregulation of cytoplasmic DNases is implicated in cytoplasmic DNA accumulation and SASP in senescent cells. Nat Commun.

[23] Yoshino M, Yoshino J, Kayser BD (2021). Nicotinamide mononucleotide increases muscle insulin sensitivity in prediabetic women. Science.

[24] Stromsdorfer KL, Yamaguchi S, Yoon MJ (2016). NAMPT-mediated NAD(+) biosynthesis in adipocytes regulates adipose tissue function and multi-organ insulin sensitivity in mice. Cell Rep.

[25] Song WL, FitzGerald GA (2013). Niacin, an old drug with a new twist. J Lipid Res.

[26] Hussain MM, Strickland DK, Bakillah A (1999). The mammalian low-density lipoprotein receptor family. Annu Rev Nutr.

[27] Wang LF, Cao Q, Wen K (2019). CD38 deficiency alleviates D-galactose-induced myocardial cell senescence through NAD+/Sirt1 signaling pathway. Front Physiol.

